# Obstructive Sleep Apnea and BMI, Body Fat Percentage in the East Asian Population: A Bidirectional and Multivariate Mendelian Randomization Study

**DOI:** 10.1002/wjo2.70100

**Published:** 2026-04-01

**Authors:** Rui Fan, Wen‐Jun Lu, Hua Zhang, Hai‐Ling Jiang, Tao Li, Yan Yan

**Affiliations:** ^1^ Department of Otorhinolaryngology Peking University Third Hospital Beijing China; ^2^ Clinical Epidemiology Research Center, Peking University Third Hospital Beijing China

**Keywords:** BMI, body fat percentage, Mendelian randomization, obstructive sleep apnea

## Abstract

**Objective:**

To explore the causal relationship between obstructive sleep apnea and body fat percentage and body mass index (BMI) in the East Asian population by using bidirectional Mendelian randomization and multivariate Mendelian randomization.

**Methods:**

We utilized genetic data on obstructive sleep apnea and BMI from the NBDC database in Japan and body fat percentage data from the Biobank in Taiwan, China. Obstructive sleep apnea cases were identified using ICD‐10 diagnostic codes rather than PSG data. Concurrently, body fat percentage and BMI were inferred from genetic variants rather than from actual body fat percentage or BMI measurements. Bivariate and multivariate Mendelian randomization analyses assessed the causal relationship between obstructive sleep apnea and obesity. The Inverse Variance Weighted method was used as the main Mendelian randomization analysis method.

**Results:**

In this study, we found that for the East Asian population (a) the forward causal relationship between body fat percentage and obstructive sleep apnea was significant (Inverse Variance Weighted: *β* 1.272, 95% CI 0.288–2.257, *p* = 0.011); (b) the forward causal relationship between BMI and obstructive sleep apnea was also significant (Inverse Variance Weighted: *β* 0.817, 95% CI 0.217–1.417, *p* = 0.008); (c) however, neither the reverse causal relationship between obstructive sleep apnea and body fat percentage (Wald ratio: *β* −0.018, 95% CI −0.041 to 0.005, *p* = 0.127) nor that between obstructive sleep apnea and BMI (Inverse Variance Weighted: *β* 0.003, 95% CI −0.021 to 0.026, *p* = 0.833) was significant. Furthermore, multivariate Mendelian randomization analyses suggested that the effect of body fat percentage on the risk of obstructive sleep apnea was independent of BMI.

**Conclusions:**

Both body fat percentage and BMI are strong risk factors for obstructive sleep apnea, and body fat percentage can function independently of BMI. Body fat percentage may be able to be used as a new indicator for screening and diagnosis of obstructive sleep apnea.

## 1 Introduction

Obstructive sleep apnea (OSA) is a clinical syndrome characterized by a series of pathophysiological changes, including sleep architecture disorders, chronic intermittent hypoxia, hypercapnia, and microarousals. These changes are caused by recurrent upper airway obstruction, leading to hypoventilation and/or respiratory interruptions during sleep [1]. According to recent studies, the global prevalence of OSA is as high as 24%, with an increasing trend year by year [2]. OSA and obesity have a complex relationship; OSA increases the risk of obesity, and obesity promotes the occurrence of OSA. That is, OSA is bidirectionally associated with obesity [3]. In addition, the prevalence of OSA in the obese population can reach up to 30% [4].

The body mass index (BMI) is the most widely used diagnostic indicator of obesity due to its simplicity and rapidity. However, body fat has been demonstrated to correlate with the development of OSA. For instance, individuals with increased neck circumference exhibit a higher probability of OSA due to the accumulation of neck fat, which results in the narrowing of the upper airway and an increased susceptibility to OSA [3]. However, BMI is not a reliable indicator of body fat. For example, male patients with OSA have a higher body fat percentage (BFP) for the same BMI than male patients without OSA [5]. Consequently, BFP emerges as a promising metric for assessing OSA risk. While clinical studies have identified associations between OSA and BFP, and BMI, these studies are not without certain limitations. Specifically, they are susceptible to confounders and reverse causality [3, 6]. To further validate and refine the results of these studies, we adopted the Mendelian randomization (MR) approach in epidemiological studies. This approach utilizes a range of genetic variants as instrumental variables (IVs) for causal inference [7]. MR studies hold considerable promise due to their potential to mitigate confounding effects, offering novel insights into the potential causal relationship between these indicators and OSA [8, 9].

Therefore, the objective of this study was to ascertain the causal relationship between OSA and BFP, as well as BMI, within an East Asian population. To this end, bidirectional MR and multivariate MR were utilized.

## 2 Methods

### 2.1 Study Design

**Figure 1:**
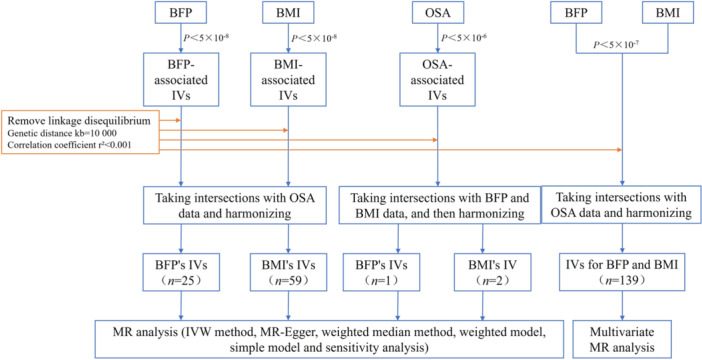
Basic flow chart of the study. The sequence of the flowchart is from left to right, and the flowchart is for BFP as exposure, BMI as exposure, OSA as exposure, and multivariate MR analysis. BFP, body fat percentage; BMI, body mass index; MR, Mendelian randomization; OSA, obstructive sleep apnea.

This study was designed as a bidirectional and multivariate MR study. The MR analysis is predicated on three underlying assumptions: (1) the assumption of correlation: IVs are strongly associated with exposure; (2) the assumption of independence: IVs are not correlated with outcome; and (3) exclusivity hypothesis: IVs do not affect outcomes through pathways other than exposure [10]. The fundamental flow chart of the study is depicted in Figure 1.

### 2.2 Data Sources

The study focused on an East Asian population, so data were selected from East Asian studies. The pooled data on OSA were obtained from a genome‐wide association study from the NBDC database in Japan, which included 473 patients with OSA and 178,337 controls [11]. The diagnosis of OSA in this study adhered to the G47.3 series as delineated in the 10th edition of the International Standard Classification of Diseases, which encompasses the patient's self‐reported symptoms, physical examination findings, and objective measurements derived from sleep recordings (i.e., an apnea‐hypopnea index [AHI] of at least five apneas per hour). The NBDC database in Japan was also utilized to obtain pooled data on BMI, which included 158,284 participants [12]. Pooled data on BMI were obtained from the Taiwan Biobank in Taiwan, China, in a study that included 102,900 participants [13].

### 2.3 Selection of Instrumental Variables

First, IVs were selected to satisfy genome‐wide significance (set to *P* < 5 × 10^−8^) and to remove linkage disequilibrium (genetic distance kb = 10AHI000, correlation coefficient *r* ^2^ < 0.001). Furthermore, it was imperative to ensure that the IVs possessed a statistical *F*‐value greater than 10 to minimize the bias that might be introduced by weak IVs [10]. Finally, the exposed IVs were harmonized with the outcome IVs, incompatible alleles and palindromic alleles with moderate allele frequencies were removed, and the final IVs obtained were used for MR analysis.

### 2.4 Bidirectional and Multivariate Mendelian Randomization Analysis and Sensitivity Analysis

The primary MR analysis methods encompass the Inverse Variance Weighted (IVW) method, the MR‐Egger method, the weighted median method, the weighted model, and the simple model method [7]. IVW is regarded as the most statistically efficacious among all the MR methods and consequently serves as the primary analysis method [7]. Concurrently, the employment of other MR methods, such as MR‐Egger, the weighted median method, the weighted model, and the simple model, increased the reliability of the results. Furthermore, Cochrane's *Q* test was utilized to analyze heterogeneity, the MR‐Egger intercept method and funnel plot were used to analyze horizontal multiplicity, the leave‐one‐out method was used to analyze whether individual IVs affected the results of the MR, and the MR‐Presso method was used to test the effect of outliers on the results [7].

### 2.5 Statistical Analysis

Statistical analysis was conducted in R 4.4.2, and results were expressed as *β*‐values and their corresponding 95% CIs. A *p*‐value less than 0.05 was considered to indicate statistical significance.

## 3 Results

### 3.1 Bidirectional Mendelian Randomization Analysis

#### 3.1.1 Results of Positive MR Analysis of BFP and OSA

**Figure 2:**
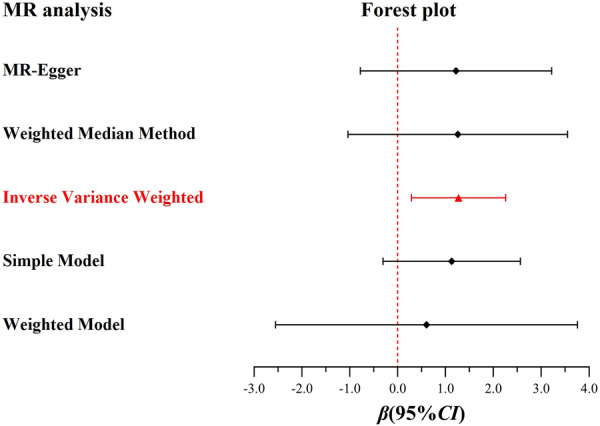
The forest plot of MR analysis results at BFP for exposure and OSA for outcome. Each row corresponds in turn to each method on the left, and red triangles indicate that the results are statistically different. BFP, body fat percentage; MR, Mendelian randomization; OSA, obstructive sleep apnea.

A total of 25 IVs independently associated with BFP were selected for positive MR analysis through rigorous screening of IVs (Supporting Information S3: Table 1). The results of IVW showed that BFP was a risk factor for OSA and that an increase in BFP could increase the risk of developing OSA (*β* 1.272, 95% CI 0.288–2.257, *p* = 0.011, Table 1). Although the results of the weighted median method, the weighted model, MR‐Egger, and the simple model did not reach statistical significance, they all showed similar causal estimates as BFP (Table 1). The forest plot of the forward MR results is shown in Figure 2.

**Table 1:** Results of Mendelian randomization analysis when BFP is exposure, and OSA is outcome (# represents *p* < 0.05).

MR methods	*β* (95% CI)	*s*	OR	*p*
MR‐Egger	0.605 (−2.548 to 3.758)	1.609	1.831	0.710
Weighted Median method	1.129 (−0.305 to 2.563)	0.732	3.093	0.123
Inverse Variance Weighted	1.272 (0.288–2.257)	0.502	3.568	0.011^#^
Simple model	1.256 (−1.039 to 3.551)	1.171	3.512	0.294
Weighted model	1.219 (−0.778 to 3.216)	1.019	3.384	0.243

Abbreviations: BFP, body fat percentage; CI, confidence interval; OR, odds ratio; OSA, obstructive sleep apnea; *s*, standard error.

#### 3.1.2 Positive Sensitivity Analysis of BFP and OSA

The MR‐Egger intercept method indicated the absence of horizontal pleiotropy in the results (*p* = 0.667), suggesting that IVs do not influence OSA through pathways other than BFP. The leave‐one‐out method indicated the stability of the results, with no individual IVs demonstrating a significant influence on the MR results. The funnel plot was largely symmetrical, and no outliers were identified using MR‐Presso. These findings collectively indicate the reliability of MR analysis and the absence of horizontal pleiotropy. The leave‐one‐out method and funnel plot for forward MR are shown in Supporting Information S1: Figure 1.

#### 3.1.3 Results of Inverse MR Analysis of BFP and OSA

Conversely, when the genome‐wide significance of the IV was set to *P* < 5 × 10^−8^, no IVs independently associated with OSA were identified through reverse MR analysis. Consequently, the genome‐wide significance was set to *P* < 5 × 10^−6^ here, identifying a single IV under these conditions (Supporting Information S4: Table 2). Due to the limited number of IVs, the Wald ratio method was employed for the MR analysis. The Wald ratio findings indicated that the causal association between the prevalence of OSA and the risk of an increase in BFR was insignificant (*β* −0.018, 95% CI −0.041 to 0.005, *p* = 0.127). Sensitivity analyses were not conducted due to the presence of a single IV.

#### 3.1.4 Results of Positive MR Analysis of BMI and OSA

**Figure 3:**
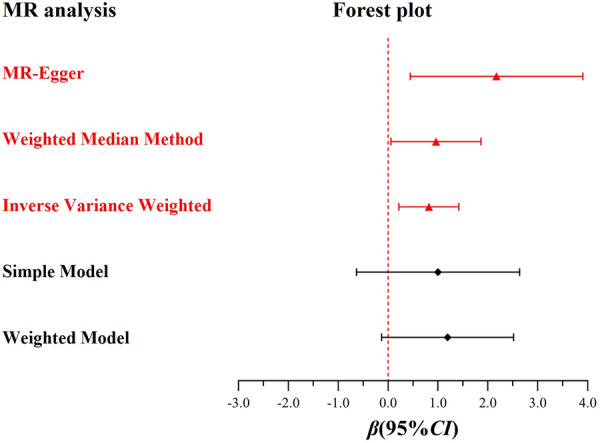
The forest plot of MR analysis results at BMI for exposure and OSA for outcome. Each row corresponds in turn to each method on the left, and red triangles indicate that the results are statistically different BMI, body mass index; MR, Mendelian randomization; OSA, obstructive sleep apnea.

A total of 59 IVs independently associated with BMI were selected for positive MR analysis through rigorous screening of IVs (Supporting Information S5: Table 3). The results of the IVW demonstrated that BMI was a risk factor for OSA and that an increase in BMI was associated with an elevated risk of developing OSA (*β* 0.817, 95% CI 0.217–1.417, *p* = 0.008, Table 2). Similar causal estimates to IVW were observed in MR‐Egger, weighted median, and weighted models, except for the Simple mode results, which were not statistically significant (Table 2). The forest plot of the forward MR results is shown in Figure 3.

**Table 2:** Results of Mendelian randomization analysis when BMI is exposure, and OSA is outcome (# represents *p* < 0.05).

MR methods	*β* (95% CI)	*s*	OR	*p*
MR‐Egger	2.173 (0.445–3.901)	0.882	8.781	0.017^#^
Weighted Median method	0.961 (0.058–1.863)	0.461	2.613	0.037^#^
Inverse Variance Weighted	0.817 (0.217–1.417)	0.306	2.263	0.008^#^
Simple model	1.001 (−0.633 to 2.636)	0.834	2.722	0.235
Weighted model	1.195 (−0.127 to 2.516)	0.674	3.302	0.082

Abbreviations: BFP, body fat percentage; CI, confidence interval; OR, odds ratio; OSA, obstructive sleep apnea; *s*, standard error.

#### 3.1.5 Positive Sensitivity Analysis of BMI and OSA

The MR‐Egger intercept method indicated the absence of horizontal pleiotropy in the results (*p* = 0.106), suggesting that IVs do not influence OSA through pathways other than BMI. The leave‐one‐out method showed stable results, with no single IV significantly influencing the MR results. The funnel plot exhibited substantial symmetry; no outliers were identified using MR‐Presso. These findings collectively indicate the reliability of MR analysis and the absence of horizontal pleiotropy. The leave‐one‐out method and funnel plot for forward MR are shown in Supporting Information S2: Figure 2.

#### 3.1.6 Results of Inverse MR Analysis of BMI and OSA

Conversely, IVs independently associated with OSA were not evaluated for reverse MR analysis when genome‐wide significance was set to *P* < 5 × 10^−8^. Consequently, the genome‐wide significance was set to *P* < 5 × 10^−6^, and 2 IVs were screened under this condition (Supporting Information S6: Table 4). The results of the IVW indicated that the causal association between the risk of OSA prevalence and increased BMI was insignificant (*β* 0.003, 95% CI −0.021 to 0.026, *p* = 0.833). Therefore, we did not perform a sensitivity analysis.

### 3.2 Results of Multivariate MR Analysis

When the genome‐wide significance was set to *P* < 5 × 10^−8^, no IVs were screened for multivariate MR analysis. Consequently, the genome‐wide significance was adjusted to *P* < 5 × 10^−7^ in this case, and 139 IVs were screened for multivariate MR analysis (Supporting Information S7: Table 5). The analysis results demonstrated that, after the effect of BMI on OSA was removed, a significant causal association between BFP and OSA remained (*β* 0.774, 95% CI 0.158–1.330, *p* = 0.013). Conversely, after removing the effect of BFP on OSA, the causal association between BMI and OSA was no longer statistically significant (*β* −0.251, 95% CI −0.708 to 0.206, *p* = 0.282). These findings suggest that BFP may independently contribute to the risk of OSA.

## 4 Discussion

In this study, we first identified that for the East Asian population, BFP was a significant risk factor for OSA (IVW: *β* 1.272, 95% CI 0.288–2.257, *p* = 0.011). Conversely, the causal association between OSA and the BFP increase was insignificant. BMI was identified as a strong risk factor for OSA (IVW: *β* 0.817, 95% CI 0.217–1.417, *p* = 0.008), consistent with previous study findings [14], yet the causal association between OSA and BMI growth was also not significant. Furthermore, multivariate MR analysis first suggested that the effect of BFP on the risk of OSA was independent of BMI. However, caution is warranted when interpreting multivariate MR results. BMI and BFP, as highly correlated phenotypes, jointly reflect overlapping characteristics of fat mass. Statistical adjustments in multivariate MR partition this shared variance. When adjusting for BMI, the fat‐specific component represented by BFP becomes isolated, yielding the observed significance. Conversely, when adjusting for BFP, the residual BMI component loses specificity, explaining the disappearance of statistical significance. These patterns likely indicate causally interconnected pathways between BFP and BMI, rather than entirely independent biological effects. Therefore, the observed BFP associations should be regarded as hypothesis‐generating findings rather than definitive evidence of independent causal effects.

A substantial body of research indicates that obesity is a major contributor to OSA. A retrospective cohort study shows that severe obesity in adolescents is significantly associated with an increase in the AHI [15]; however, a meta‐analysis shows that bariatric surgery is effective in alleviating OSA [16]. The underlying pathophysiology of obesity‐induced OSA remains to be fully elucidated, though potential mechanisms may involve the effects of fat deposition on airway anatomy or alterations in upper airway function [17]. For instance, a retrospective study utilizes x‐ray scans to assess body fat distribution in children, revealing a significant association between neck fat distribution and the AHI [18].

BMI is the most widely used diagnostic indicator of obesity and has been extensively utilized in epidemiologic studies [19]. However, it does not fully demonstrate body fat content. Furthermore, the correlation between BMI and body fat is subject to variation based on gender, age, and racial ethnicity, resulting in discrepancies in the representation of body fat across different BMI categories [20]. Consequently, BMI is not a universally applicable indicator for assessing OSA risk, particularly in obese patients [20]. Thus, it is necessary to find a new metric that can be used to predict OSA risk. BFP, defined as the proportion of body fat in an individual's total body weight, has been identified as a reliable indicator of the body's fat amount. Our study identified a significant positive causal relationship between BFP and OSA. However, our findings are based on genetic markers rather than actual measured BFP, and OSA was defined solely by ICD codes without utilizing polysomnography‐confirmed AHI values. Therefore, this study provides only genetic evidence suggesting that higher BFP causally increases OSA risk and may be conceptually superior to BMI as a biomarker. However, our research cannot validate BFP as a clinical screening marker for OSA, as this requires validation through larger scale phenotyping studies.

Our study possesses several noteworthy merits. Primarily, MR's theoretical framework precludes confounding bias and controls for the potential interference of reverse causality on outcomes, thereby minimizing the impact of confounders and reverse causality, thus enhancing the reliability of results. Moreover, our study pioneeringly analyzes the causal relationship between the OSA and BFP, and BMI, by employing MR based on GWAS data from an East Asian population, a significant contribution within the broader field. Finally, employing multiple MR methodologies, in conjunction with IVW and a thorough quality assessment of IV, fortifies the credibility of the study's findings.

Furthermore, OSA identification based on ICD codes lacks stratification by severity (e.g., AHI levels), which may introduce misclassification bias. When extrapolating these findings to specific comorbidities or complications, caution regarding their reliability is warranted. For instance, moderate and severe OSA, rather than mild OSA, are more closely associated with cardiovascular events. Relying solely on OSA's ICD coding categorizes all mild, moderate, and severe cases into a single group. Consequently, extrapolating this approach to actual patient cardiovascular outcomes is suboptimal, representing one of the study's primary limitations.

However, the study has several limitations: First, the lack of comprehensive GWAS data limited our ability to conduct subgroup analyses based on variables such as sex, geographic location, and age. This limitation restricts the applicability and generalizability of our findings. Second, the reliance on data primarily originating from East Asian populations restricts the extrapolation of findings to other ancestral groups with different fat distribution or airway anatomy, considering the documented disparities in gene expression and genetic susceptibility among different racial groups. For instance, East Asian populations exhibit comparatively lower BMI and BFP compared to white populations, and there are some discrepancies in the prevalence of OSA. This study focuses on the East Asian population, and we must consider its impact when examining the causal relationship between OSA and BFP, and BMI in the East Asian population. Additionally, although all GWAS data represent East Asian populations, differences in allele frequencies and linkage disequilibrium structures across subpopulations may still affect the universality of IV efficacy, MR assumptions, and causal estimates. Therefore, the presence of genetic heterogeneity cannot be ruled out. Future validation studies in genetically harmonized or ancestrally matched cohorts are needed to confirm the reliability of these findings. Third, the BFP measurement method employed in the genetic database is bioelectrical impedance analysis, not dual‐energy x‐ray absorptiometry. Consequently, this may introduce a certain inaccuracy into the analysis results. Fourth, the study's findings are constrained by the dearth of IV data in East Asia, particularly in scenarios where OSA is the exposure and BMI or BFP is the outcome. The number of IVs obtained is found to be quite limited in such instances. This limitation prevents subsequent sensitivity analyses, which may reveal omission bias. Fifth, the results of sample overlap analysis suggest the possibility of sample overlap between the OSA and BMI GWAS datasets (Supporting Information S8: Table 6), which may lead to biased causal estimates and undermine the validity of MR analysis. Finally, in the inverse MR analysis, the IV for OSA was derived from a dataset containing only 473 OSA cases, limiting the statistical power to detect causal effects. Therefore, estimates from the inverse analysis should be interpreted with caution. These findings should be regarded as preliminary results and require validation in larger cohorts with sufficient statistical power to reliably assess potential inverse causality. To address these issues and enhance the robustness of our findings, future studies should incorporate diverse GWAS datasets encompassing various populations and ethnicities to validate and broaden our findings.

## 5 Conclusion

Our study found that BFP and BMI are important risk factors for OSA and that BFP can act independently of BMI. Therefore, BFP may be a more sensitive indicator than BMI.

## Author Contributions

**Rui Fan:** conceptualization, investigation, writing – original draft. **Wen‐Jun Lu:** investigation, data curation, writing – original draft. **Hua Zhang:** investigation, formal analysis, methodology. **Hai‐Ling Jiang:** investigation, resources, writing – original draft. **Tao Li:** conceptualization, funding acquisition, writing – review and editing. **Yan Yan:** conceptualization, project administration, writing – review and editing.

## Ethics Statement

This study utilized only publicly available GWAS abstract data and therefore did not require ethical approval.

## Conflicts of Interest

The authors declare no conflicts of interest.

## Supporting information


**Supplementary Figure 1:** BFP as exposure, leave‐one‐out method (a), and funnel plot (b) for sensitivity analysis (***s*** **:** standard error). Abbreviation: BFP, body fat percentage.

**Supplementary Figure 2:** BMI as exposure, leave‐one‐out method (a), and funnel plot (b) for sensitivity analysis. Abbreviation: BMI, body mass index.

**Supplementary Table 1:** Positive MR analysis of BFP and OSA. Abbreviations: BFP, body fat percentage; MR, Mendelian randomization; OSA, obstructive sleep apnea.

**Supplementary Table 2:** Inverse MR analysis of BFP and OSA. Abbreviations: BFP, body fat percentage; MR, Mendelian randomization; OSA, obstructive sleep apnea.

**Supplementary Table 3:** Positive MR analysis of BMI and OSA. Abbreviations: BMI, body mass index; MR, Mendelian randomization; OSA, obstructive sleep apnea.

**Supplementary Table 4:** Inverse MR analysis of BMI and OSA. Abbreviations: BMI, body mass index; MR, Mendelian randomization; OSA, obstructive sleep apnea.

**Supplementary Table 5:** Multivariate MR analysis (*P* < 5 × 10^−7^). Abbreviation: MR: Mendelian randomization.

**Supplementary Table 6:** Analysis of sample overlap between BMI and OSA. Abbreviations: BMI, body mass index; OSA, obstructive sleep apnea.

## Data Availability

The data that support the findings of this study are available on request from the corresponding author.
