# Supplementary figures and images for: Obstructive Sleep Apnea and BMI, Body Fat Percentage in the East Asian Population: A Bidirectional and Multivariate Mendelian Randomization Study
Source: World J Otorhinolaryngol Head Neck Surg. 2026 Apr 1:10.1002/wjo2.70100. Online ahead of print. doi: 10.1002/wjo2.70100 (PMC13399181; doi:10.1002/wjo2.70100)

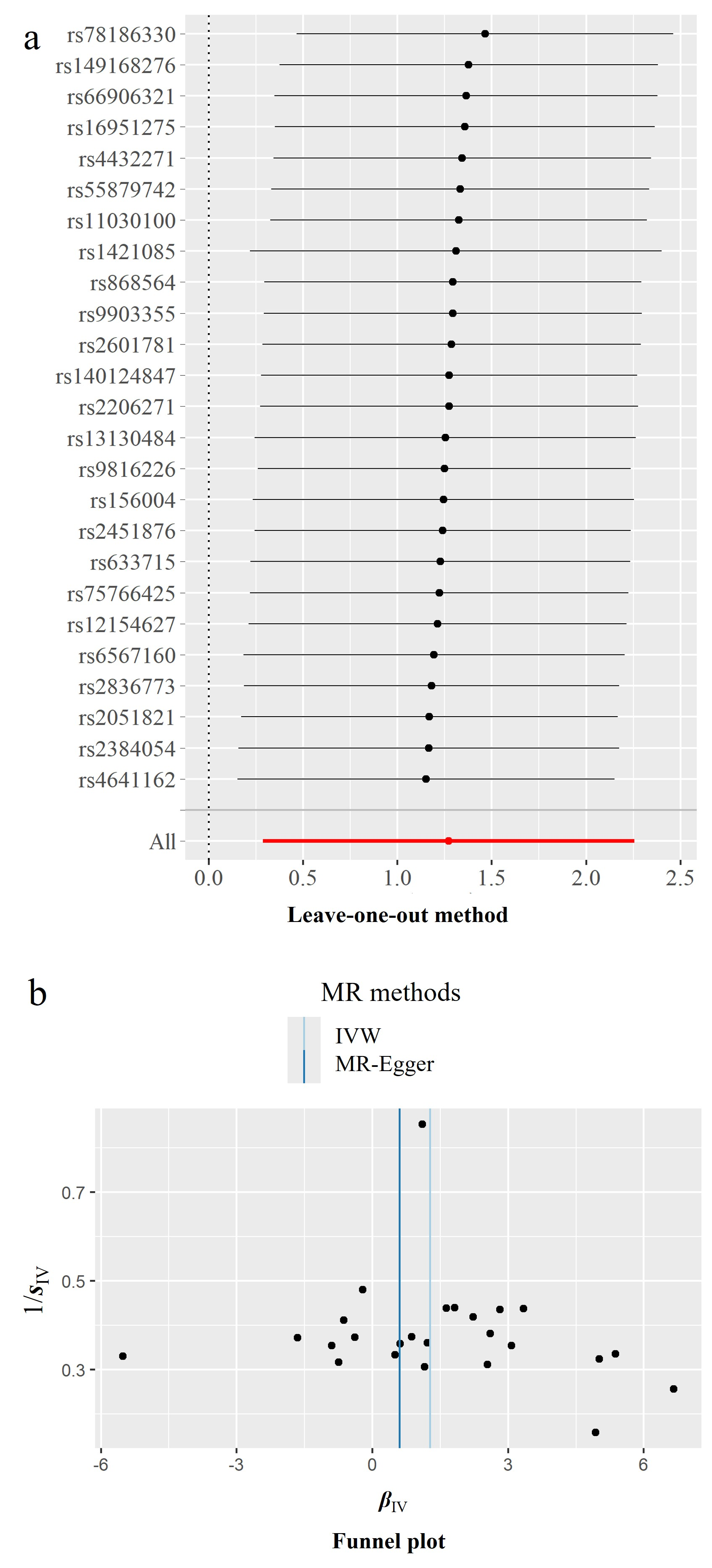

Supplement: Supplementary file 1 — Supplementary Figure 1: BFP as exposure, leave‐one‐out method (a), and funnel plot (b) for sensitivity analysis ( s : standard error). Abbreviation: BFP, body fat percentage. [file WJO2-9999-0-s001.tiff]

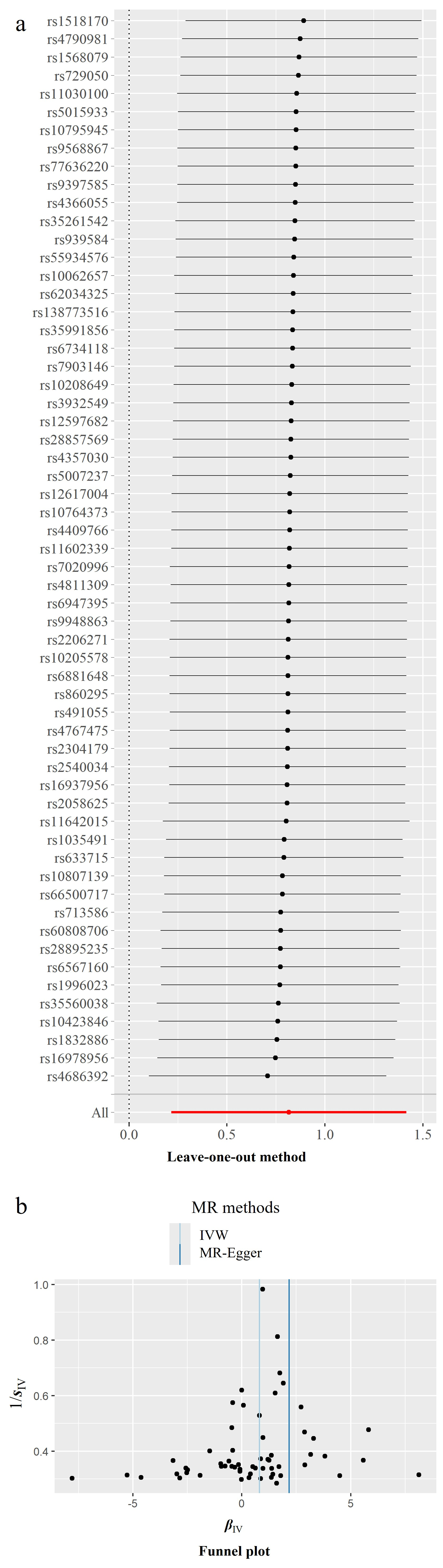

Supplement: Supplementary file 2 — Supplementary Figure 2: BMI as exposure, leave‐one‐out method (a), and funnel plot (b) for sensitivity analysis. Abbreviation: BMI, body mass index. [file WJO2-9999-0-s005.tiff]
